# Prediction of screw loosening by measuring the insertion torque in non-osteoporotic patients: an in vitro study

**DOI:** 10.1186/s12891-025-08654-4

**Published:** 2025-04-25

**Authors:** Jan Ulrich Jansen, Laura Zengerle, Carsten Hackenbroch, Jens Dreyhaupt, Youping Tao, Hans-Joachim Wilke

**Affiliations:** 1 Institute of Orthopaedic Research and Biomechanics, Centre for Trauma Research Ulm, Ulm University Medical Centre, Helmholtzstraße 14, 89081 Ulm, Germany; 2 Department of Radiology, German Armed Forces Hospital Ulm, Oberer Eselsberg 40, 89081 Ulm, Germany; 3 Institute for Epidemiology and Medical Biometry, Ulm University, Schwabstraße 13, 89075 Ulm, Germany

**Keywords:** Insertion torque, Pedicle screw, Distraction, Scoliosis, Screw loosening, Biomechanics

## Abstract

**Background:**

Pedicle screws are commonly used in spinal surgeries, but screw loosening remains a major concern, even in non-osteoporotic patients. Predicting pedicle screw stability via the insertion torque is a controversial topic, mainly studied on osteoporotic cadavers. Whether the insertion torque is suitable for patients with healthy bone mineral density (BMD) remains unknown. The aim was to investigate the influencing factors, namely insertion torque, BMD, screw diameter, length, surface area, volume, screw-in rotations, vertebral level, on the screw loosening stability during distractions and to understand if intra-operative predictions are possible.

**Methods:**

Non-osteoporotic thoraco-lumbar vertebrae (*n* = 50) were used to implant five different pedicle screws (*n* = 100) while measuring the insertion torque. After embedding the endplates, the force needed to distract the screw head by 1 mm was tested.

**Results:**

The insertion toque (2.3 ± 0.9 Nm) showed the highest influence on the distraction force (324.8 ± 84.4 N) followed by the screw size and vertebral level. BMD did not show any effects.

**Conclusions:**

The linear correlation of insertion torque and the bending force suggests an alternative prediction metric for screw loosening which could improve the outcome of surgeries and patients’ safety. This is potentially a simple, intra-operative method, which can be used in future.

**Supplementary Information:**

The online version contains supplementary material available at 10.1186/s12891-025-08654-4.

## Background

To date, the pedicle screw remains the gold standard for spinal fixation. Over the last two decades, it has gained worldwide acceptance as part of a construct using plates, rods and wires for spinal instrumentation [1–3]. They are inserted postero-anteriorly into the pedicle of the vertebra during surgery and allow force transmission between the vertebral bone and the instrumentation. However, the use of pedicle screws is not without complications, with screw loosening being a frequent issue [4]. Osteoporosis, a disease characterized by reduced bone mineral density (BMD), plays a significant role in these complications by decreasing bone stiffness and structural integrity, often worsening with age [4–10]. Studies show that screw loosening occurs in up to 60%, osteoporotic patients, compared to just 1–15% in non-osteoporotic patients [4].

Consequently, screw loosening is a concern even in young patients. These are often non-osteoporotic individuals with traumatic injuries or adolescent idiopathic scoliosis [11–14], which often have to undergo repeated interventions [15]. Also in these patients, a key challenge is determining screw stability and predicting loosening to ensure optimal surgical outcomes. For this reason, many studies have investigated the insertion torque of pedicle screws and its resulting potential for predicting pedicle screw stability and loosening [16–26].

While early studies, such as those by Zdeblick et al. in 1993, suggested that insertion torque could predict bone-metal interface failure and screw loosening [26], more recent studies have produced conflicting results. For example, Kwok et al. found variable correlations between insertion torque and pullout force, questioning its predictive value [24]. Similarly, Ozawa et al. and Okuyama et al. observed associations between insertion torque and osteoporosis grade but concluded that insertion torque alone was not a reliable predictor of screw loosening or clinical outcomes [16, 20, 23]. Other research has shown that the insertion torque can offer useful predictions, particularly in biomechanical stability, with studies by Carmouche et al. and Weidling et al. demonstrating promising correlations with screw pull-out forces in cadaveric and synthetic models [18, 19, 21, 24, 27].

Despite these inconsistencies, most studies have focused primarily on osteoporotic vertebrae, with limited research on non-osteoporotic conditions, such as those found in younger patients. Furthermore, many previous biomechanical testing methods, such as pull-out tests, may not fully replicate the physiological loading experienced by pedicle screws [28, 29]. Bending tests are the preferred test method compared to pull-out tests since pedicle screws are typically loaded physiologically in the cranio-caudal direction and loosen due to the so-called windshield wiper effect [28–31]. Additionally, factors such as screw geometry, BMD, and the influence of insertion torque on screw stability are still not fully understood.

In light of these challenges, the purpose of this in vitro experiment was to investigate the association of screw insertion torque with a distraction force necessary to create screw loosening in non-osteoporotic vertebrae using a sufficient data collective. By evaluating additional factors such as BMD, screw geometry, screw-in rotations, vertebral level, surgical side left/right, we hope to further elucidate the biomechanical behavior of pedicle screws in non-osteoporotic conditions. Ultimately, understanding these relationships could lead to improved surgical techniques and better patient outcomes.

## Materials and methods

### Specimens and Preparation

**Fig. 1:**
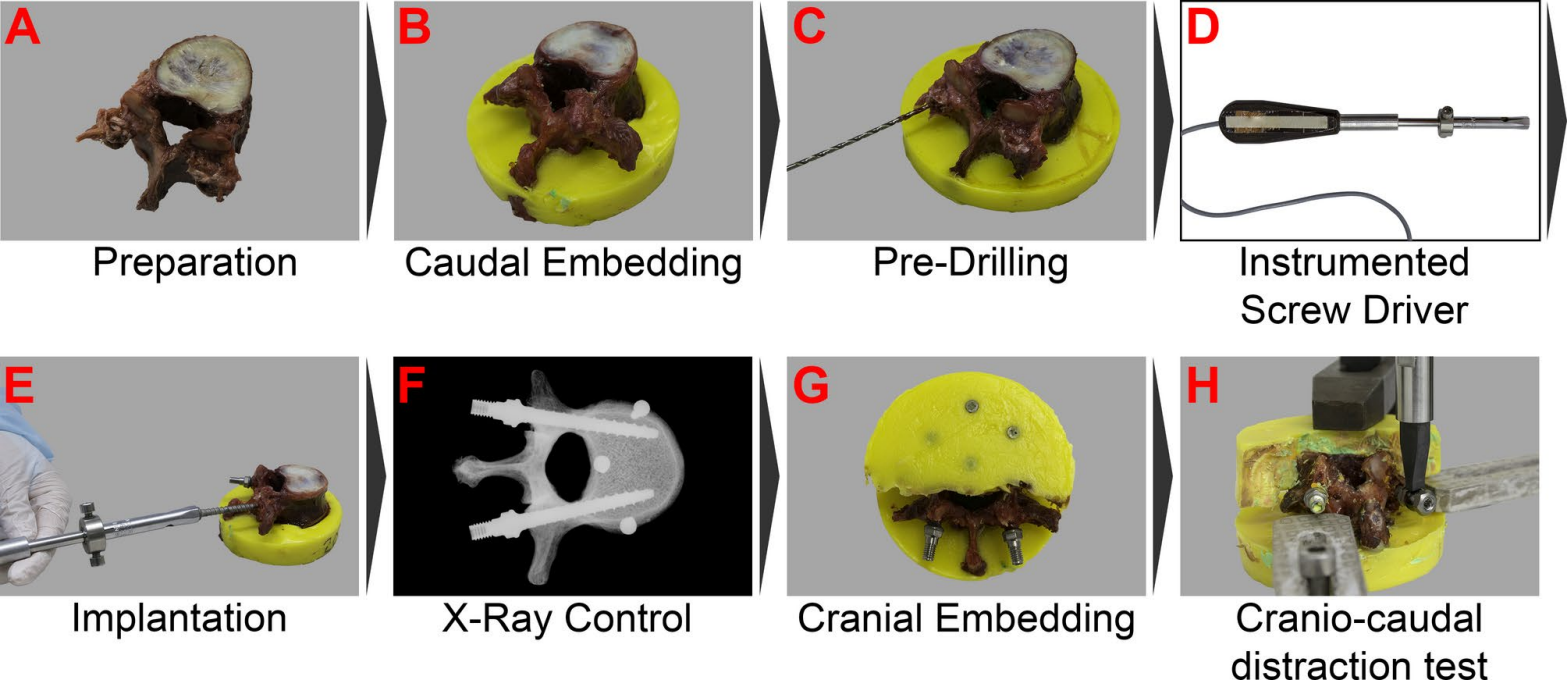
Experimental steps chronologically from A to H: **(A)** Preparation of single vertebral body; **(B)** Embedding of caudal side of vertebral body; **(C)** Pre-drilling with 2.5 mm; **(D)** Image of instrumented screwdriver used to implant the pedicle screws and measure insertion torque; **(E)** Implantation of the pedicle screws starting with left pedicle; **(F)** Example for X-ray control; **(G)** Embedding of the cranial side of vertebral body leaving enough space for subsequent biomechanical testing; **(H)** Distraction test in materials testing machine simulating distraction in cranio-caudal direction during surgery

A total of 50 single vertebrae from 14 donors were used from level T9 to L4 (10 male, 4 female) (Table 1). Only vertebrae from donors under the age of 50 years (mean donor age: 40.9 ± 8.3 y) and with a BMD of the individual vertebra of more than 120 mg(Ca-HA)/cm^3^ were included. Ethical approval for usage of the specimens was given by the ethical committee board of the University of Ulm (No. 298/19). The specimens were acquired from body donation programs, where legally valid informed consent was obtained from all participants and/or their legal guardian(s) (Science Care Inc., Phoenix, USA). All methods were performed in accordance with relevant guidelines and regulations and following the Declaration of Helsinki. The BMD of the cadaveric spines was determined with quantitative computed tomography (qCT) for every single vertebra (SOMATOM Definition AS+, Siemens, Germany). After removing all soft tissue of the spines including ligaments and tendons, the individual vertebrae were separated. Hereby, the discs were removed while the cartilaginous endplate was left intact (Fig. 1A). Afterwards, the caudal endplates of the specimens were embedded into Polymethylmethacrylate (PMMA) (Technovit, Kulzer, Wehrheim, Germany) using self-tapping universal screws for improved anchoring of the specimen in the PMMA (Fig. 1B). The specimens were stored at -25 °C in triple-sealed foil bags (also for qCT) and thawing was conducted gentle over night for 10 h under cool conditions (2 °C).

**Table 1:** Specimen overview

Donor	Age	Segments
1	37	T11-L4
2	36	T10-L1
3	32	L2
4	41	L2-L4
5	49	T9-L4
6	41	T10-T11
7	43	L1
8	38	T9-L1
9	49	T9-L4
10	19	T11-L1
11	50	T10-L4
12	45	T9
13	47	T9
14	46	T9; L2-L4

Detailed information about tested specimens used for the investigation of the insertion torque as a prediction tool for screw loosening in young patients.

### Screw insertion with torque measurement

**Fig. 2:**
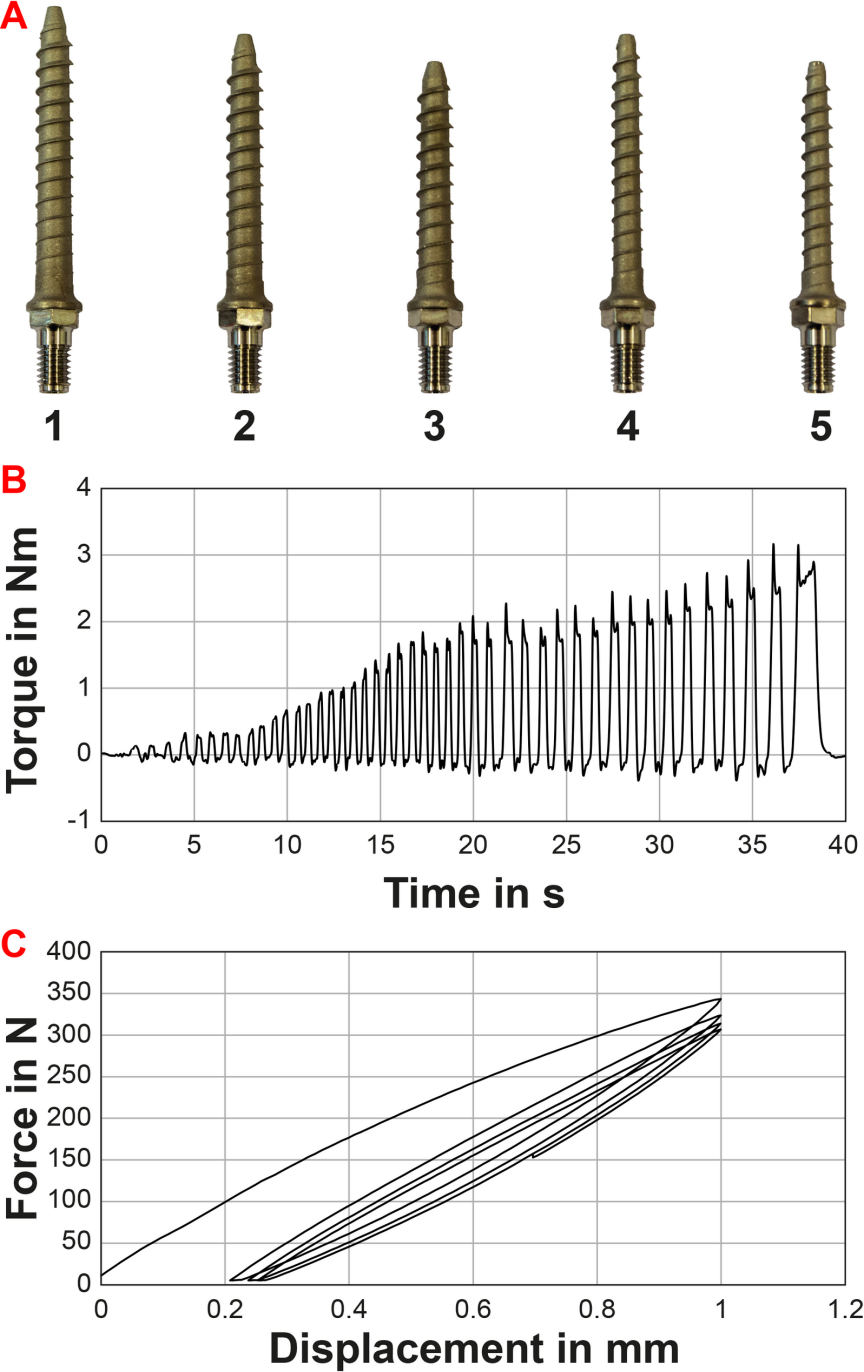
Further methodological insights and example curves: **A)** Five different pedicle screw with different diameter and length in mm used for the experiment: (1) 6.5 × 50, (2) 6.5 × 45, (3) 6.5 × 40, (4) 5.5 × 45, (5) 5.5 × 40; **B)** Exemplary curve of the insertion torque over time showing the series of manual rotations by the operator. This was used to determine the maximum insertion torque. **C)** Exemplary plot of displacement-force-diagram for distraction test visualizing the four subsequent loadings and the resulting setting process

The pedicle screws were provided by Apifix Ltd. (Yokneam Illit, Israel) and selected individually for each vertebra by an experienced spine surgeon (right-handed). The length, diameter, surface and volume of the pedicle screws were determined from the screw geometry via computer-aided design files. Five different screw sizes were available (Fig. 2A). In the selection process, the biggest possible diameter and length were prioritized. The pedicle size represents the limiting anatomical size for the pedicle screw diameter in practice and this selection criterion reflected the most difficult case and standardized the selection [21]. Perforation of the screw tip anteriorly out of the vertebral body was prevented by suitable screw lengths and did not occur. A screwdriver, equipped with strain gauges, was used to measure the insertion torque on the screw while inserting it into the vertebra (Fig. 1D). The calibration of the screw driver was performed before starting a test series. Before insertion, holes with a diameter of 2.5 mm and a depth of 20 mm were pre-drilled for all screw types (Fig. 1C). The screws were inserted into both pedicles of every specimen (Fig. 1E). The screws were inserted into the left pedicle first. During insertion, the screwing rotations were counted and the insertion torque continuously measured (Fig. 2B). The number of screw rotations were obtained by a lab assistant observing the screw driver. The peaks in example Fig. 2B correspond rather to half rotations during insertion and were not used for evaluating rotations. Screw insertion was stopped 1 mm before contact between the screw head and the pedicle to avoid distorting torque peaks measured (Fig. 1G). The insertion torque was defined as the maximum torque during insertion. Appropriate placement and alignment of the screw was checked after implantation by X-ray control (Fig. 1F). If not achieved, the results were not included.

**Table 2:** Detailed geometrical data about the five different types of screws implanted

Screw	Diameter in mm	Length in mm	Surface area in mm^2^	Volume in mm^3^	Number of uses
1	5.5	40	676.2	664.1	18
2	5.5	45	769.7	748.3	40
3	6.5	40	829.2	843.8	6
4	6.5	45	936.8	955.8	18
5	6.5	50	1043.4	1076.2	18

*mm* millimeters

### Distraction test with materials testing machine

Pedicle screws are not loosened physiologically by pullout forces but by bending forces. In order to simulate these bending moments, we applied a “distraction force” on the screw head that represented the loading scenario on the screw head. The distraction force was defined as the force required to displace the pedicle screw head by 1 mm in the cranio-caudal direction. After implanting the screws, the cranial anterior part of the vertebrae was embedded (Fig. 1G). Self-tapping screws were also used for better connection of PMMA and vertebral body. The posterior column including the inserted pedicle screw heads were kept free of PMMA (Fig. 1G). Then, an interoperative distraction of 1 mm was simulated using a universal testing machine (Zwick Z010, Ulm, Germany) under displacement control. An axial displacement of 1 mm was applied centrically to the sphere of the ball joint by a flat rectangular indenter (4 mm x 8 mm) (Fig. 1H) and the resultant axial force was measured by a 1 kN load cell. Testing was performed with a pre-load of 5 N, a speed of 10 mm/min, and multiple cycles. After 4 cycles of loading, the test was manually stopped. The 3rd cycle was used for further evaluation since the 4th cycle was used to ensure that the viscoelastic setting process was complete (Example in Fig. 2C) [32].

### X-ray

Conventional X-rays were conducted with 56 keV and 2 mAs in a combination of a cabinet x-ray system (Faxitron, Hewlett-Packard Co, McMinnville, USA) and a digital radiography panel (PIXX1417, PIXXGEN Corporation, Korea) for image digitalization. X-rays were taken in the intact state of all specimens prior to and after the bending test in antero-posterior and latero-lateral perspective. With the help of flanges, reproducible positions between the states pre and post testing could be realized.

### Data collection and statistics

The data were collected and processed with Excel (Microsoft 16.88, Redmond, USA) and afterwards analyzed with SPSS 29 (IBM, Armonk, USA) and SAS, version 9.4 (SAS Institute, Cary, NC) under Windows. Continuous data are described using mean and standard deviation. In addition, minimum and maximum are provided. Categorical data are presented as frequencies and percentages. The effects of potential influencing variables (BMD, side right/left, insertion torque, screw diameter, screw length, screw volume, screw-in rotations, vertebral level) on the target variable distraction force were first determined using univariate linear mixed effects regression models. In a second step, a multiple linear mixed effects regression model was applied to the data. The significance level was 0.05 (two-sided) for all tests. Due to the explorative nature of this study, all results from statistical tests have to be interpreted as hypothesis-generating. Adjustment for multiple testing was not made.

## Results

Non-osteoporotic bone quality for every single vertebra was ensured by qCT leading to a mean BMD of 149.5 ± 29.5 mg(Ca-HA)/cm^3^. Using an instrumented screwdriver, the insertion torque was measured during implantation of the pedicle screws (*n* = 100). After X-ray control, the distraction force required to deflect the screw head cranio-caudally by 1 mm was measured. Different influencing factors were analyzed. The influences on the insertion torque were examined first to better understand the effects on the target “distraction force”.

### Impact of surgical side (left/right)

Separate univariate linear mixed effects regression models were used for analyzing the influences of surgical side (left or right pedicle), vertebral level (T9-L4), screw type (1–5, Fig. 2A), screw surface, screw volume, screw length, screw diameter, screwdriver rotations, and BMD on the insertion torque. Initially, the influence of the surgeon was checked by comparing the insertion torque differences between the left and right sides. For all influencing factors except the BMD, the surgical side played a significant role for the outcome (*p* < 0.05). The absolute differences for the insertion torque were 0.35 ± 0.42 Nm and for the distraction force 58.5 ± 45.5 N. This was taken into account in all further calculations and evaluations.

### Influences on the insertion torque

For the insertion of the pedicle screws, the torques ranged at a mean of 2.3 ± 0.9 Nm (min: 0.9, max: 5.7 Nm) while needing a mean number of screwdriver rotations of 12.2 ± 1.8 (min: 7, max: 17). The insertion torque was dependent on the vertebral level (*p* < 0.01) and increased from the thoracic to the lumbar vertebrae: 1.8 ± 0.6 Nm for T9, 1.5 ± 0.3 Nm for T10, 1.7 ± 0.4 Nm for T11, 1.7 ± 0.4 Nm for T12, 2.5 ± 0.8 Nm for L1, 2.7 ± 0.8 Nm for L2, 3.0 ± 0.6 Nm for L3, and 3.6 ± 0.9 Nm for L4 (*n* ≥ 12). The screw type (i.e. whether screw 1, 2, 3, 4, or 5, Fig. 2A) as well as the screw properties– surface, volume, length, and diameter– had an influence on the insertion torque (*p* < 0.01). The torque was for screw type 1: 1.6 ± 0.5 Nm, for 2: 1.7 ± 0.3 Nm, for 3: 2.4 ± 0.5 Nm, for 4: 2.9 ± 0.7 Nm, and for 5: 3.5 ± 0.8 Nm (group sizes: Table 2). Using the geometrical information from Table 2, it followed that the insertion torques also increased with screw surface and volume (*p* < 0.01). The insertion torque also increased with screw length and diameter, namely a torque of 1.8 ± 0.6 Nm was found for 40 mm length, 2.1 ± 0.7 Nm for 45 mm length, 3.5 ± 0.8 Nm for 50 mm length, 1.7 ± 0.4 Nm for 5.5 mm diameter, and 3.1 ± 0.8 Nm for 6.5 mm diameter, respectively. With increasing number of screwdriver rotations k, the insertion torque increased: 7 ≤ k ≤ 10: 1.6 ± 0.6 Nm; 10 < k ≤ 11: 1.7 ± 0.3 Nm; 11 < k ≤ 12: 2.0 ± 0.6 Nm; 12 < k ≤ 13: 2.6 ± 0.7 Nm; 13 < k ≤ 14: 2.9 ± 1.1 Nm; 14 < k ≤ 17: 3.6 ± 0.9 Nm (*p* < 0.01). The BMD had no influence on the insertion torque for the data collective used in this study (*p* = 0.93).

### Influence on the distraction force

**Fig. 3:**
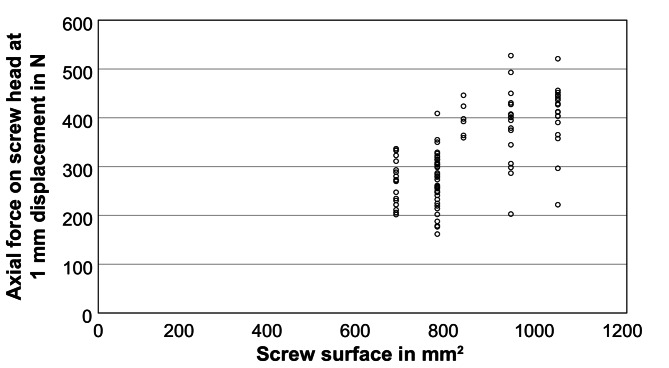
Distraction force (representing the screw loosening risk) at 1 mm displacement depending on the screw surface area. Every data point represents one inserted pedicle screw (*n* = 100)

**Fig. 4:**
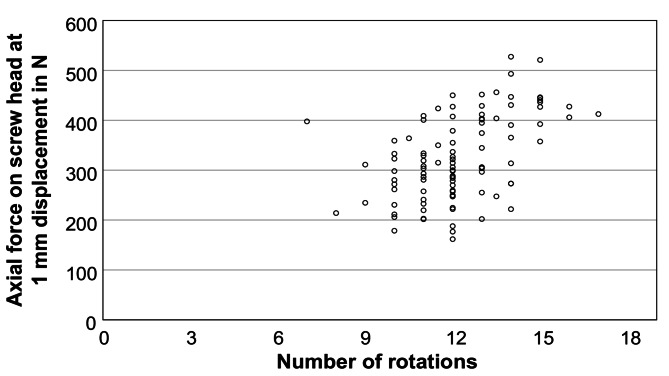
Distraction force (representing the screw loosening risk) at 1 mm displacement depending on the number of rotations needed for insertion of the screw. Every data point represents one inserted pedicle screw (*n* = 100)

**Fig. 5:**
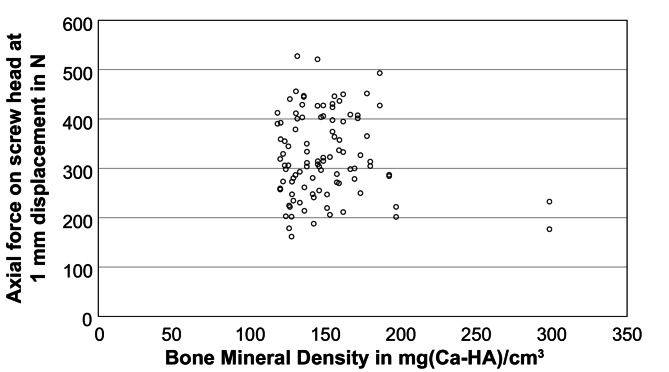
Distraction force (representing the screw loosening risk) at 1 mm displacement depending on the bone mineral density (BMD) in mg(Ca-HA)/cm^3^. Point cloud shows non-osteoporotic BMD for all specimens and no statistical effect of the BMD on the distraction force within this data collective

**Fig. 6:**
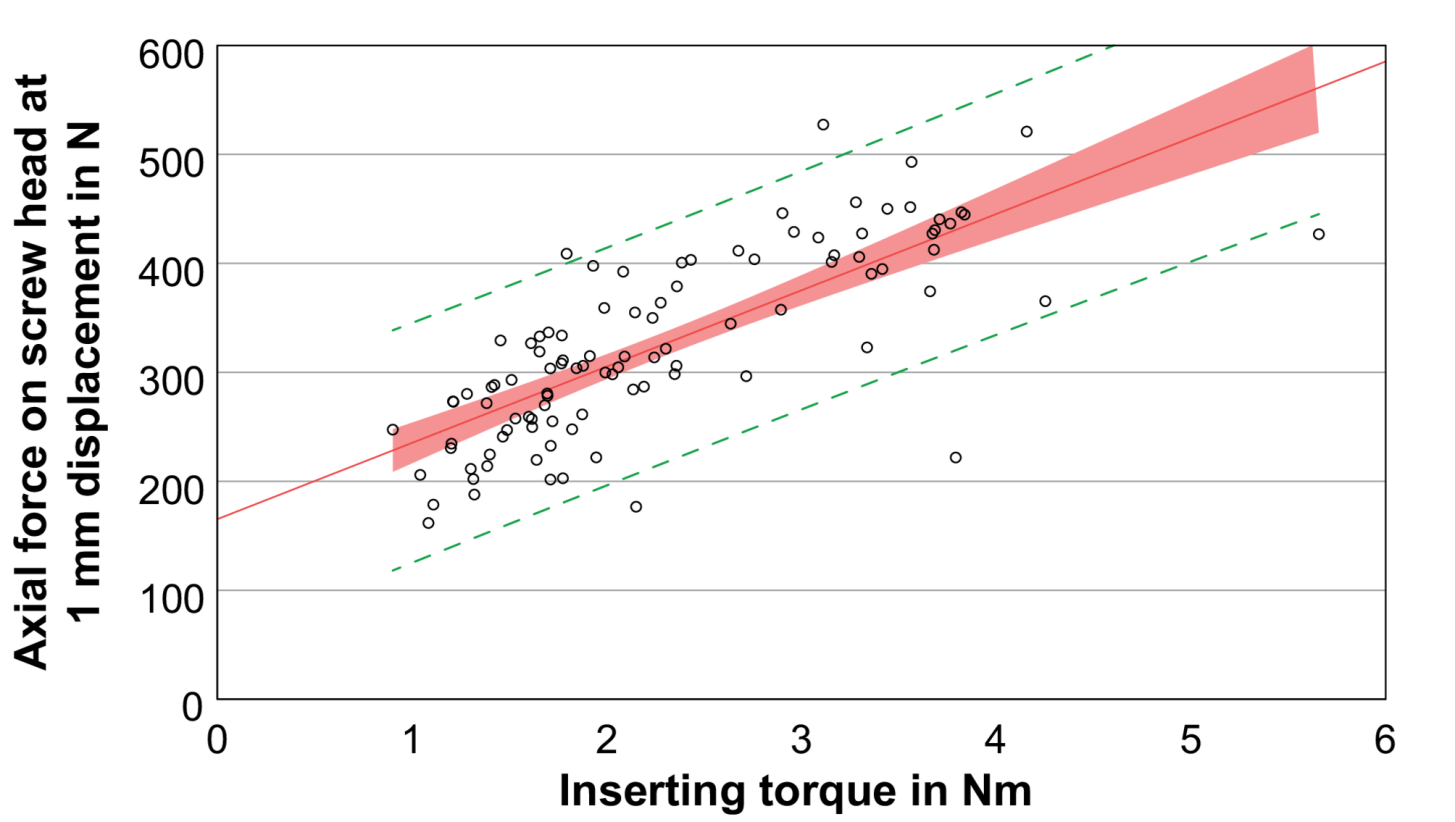
Distraction force (representing the screw loosening risk) at 1 mm displacement depending on the inserting torque. Red line indicates linear regression between insertion torque and bending force for all 100 tested pedicle screws. The 95% confidence interval of the regression (green dashed lines) and the 95% confidence limits of the regression line (rendered in solid light red) are shown

After implantation of the screws and embedding of the cranial side of the vertebral body, the distraction force for a dislocation of 1 mm was determined in four repetitions, using the third repetition for the evaluation. The distraction force for all screws amounted to 324.8 ± 84.4 N with a maximum and minimum value of 527.4 N and 161.7 N, respectively. A univariate and multivariate model was used to determine the influence of surgical side, vertebral level, screw type (1–5), screw surface, screw volume, screw length, screw diameter, screwdriver rotations, BMD, and insertion torque. The normal distribution and the statistical requirements of the target variable, i.e. the distraction force, was confirmed beforehand. Furthermore, the surgical side had no influence on the target variable of distraction force. The vertebral level had a significant effect on distraction force (*p* < 0.01) and showed a trend of increasing possible distraction forces from thoracic (T9) to lumbar vertebrae (L4): 296.3 ± 51.9 N for T9, 248.3 ± 46.9 N for T10, 262.0 ± 52.1 N for T11, 266.4 ± 45.0 N for T12, 361.7 ± 72.0 N for L1, 364.4 ± 68.5 N for L2, 393.7 ± 54.2 N for L3, and 426.4 ± 65.7 N for L4 (*n* ≥ 12). Similar to the insertion torque, there was a significant influence on the distraction force for all screw parameters (*p* < 0.01). For the screw types 1 to 5 including the associated screw surfaces and volumes (Table 2), the distraction force was: for 1: 270.4 ± 45.9 N, for 2: 273.9 ± 53.3 N, for 3: 397.2 ± 33.7 N, for 4: 386.5 ± 77.4 N, and for 5: 406.4 ± 66.1 N (exemplary Fig. 3). The distraction force increased with screw length and diameter. Regarding the screw length, the smallest force was obtained for 40 mm with 302.1 ± 70.3 N, followed by 45 mm with 308.8 ± 80.6 N, and by 50 mm with 406.4 ± 66.1 N. Regarding the influence of the diameter, a force of 272.8 ± 50.8 N was found for 5.5 mm and 396.6 ± 67.2 N for 6.5 mm, respectively. The number of insertion rotations showed an increasing relationship similar to the insertion torque (Fig. 4, *p* < 0.01). The BMD had no influence on the distraction force for the data collective used in this study (Fig. 5, *p* = 0.25). In order to avoid any collinearities and sequencing effects, the influence of the insertion torque on the distraction force was determined using a multiple mixed linear regression model including influences of screw type and vertebral level, which found a significant linear regression for the insertion torque (Fig. 6, *p* < 0.01).

## Discussion

This study aimed to investigate the influence of several factors– such as insertion torque, BMD, screw geometry, screw-in rotations, vertebral level, and surgical side (left/right)– on the intraoperative distraction stability and the primary loosening behavior of pedicle screws in the thoraco-lumbar spine in vitro. Among these factors, insertion torque emerged as the strongest predictor for the magnitude of the distraction force required to induce 1 mm displacement. In particular, the measurements could demonstrate– for non-osteoporotic, healthy vertebrae– a linear relationship between insertion torque and distraction force. This relationship between pedicle screw insertion torque and pedicle screw stability, i.e. risk of screw loosening, has been of interest since a long time to predict surgery outcome [16, 18–20, 22, 23, 28] and to decide on the need of additional actions, such as detrimental bone cement usage [33]. To our knowledge, this is the first study with a large sample size of non-osteoporotic vertebrae that also examines additional parameters, including the influence of vertebral level, screw geometry, screwdriver rotations and surgical side. These findings may also help in the development of new implants and numerical methods in the future.

Reduced BMD is one of the certain risk factors for screw loosening and several other postoperative complications [34]. Various reviews have reported a relationship between BMD and mechanical strength of the screw-bone-interface [4, 34, 35]. Interestingly, with a BMD ≥ 120 mg(Ca-HA)/cm^3^ for all specimens, no correlation for the insertion torque and the distraction force is determined in the present study (Fig. 5). Thus, it can be assumed that above a certain minimum BMD, no influence of the BMD on the insertion torque or the stability of the screw can be observed. This threshold has been identified by studies as directly corresponding to the threshold for osteoporosis, i.e. 80 mg(Ca-HA)/cm3, and conversely, pedicle screw augmentation in non-osteoporotic bone does not result in further stability improvement [6, 36, 37]. The absolute values for the insertion torque and the distraction force cannot be compared directly with the literature but are reasonable. Firstly, our study includes the BMD of each individual vertebral body using the more precise qCT in contrast to many comparative studies with dual-energy X-ray absorptiometry (DEXA). And unfortunately, DEXA is limited by not taking the bone volume into account leading to a lack of granularity and an overestimation of BMD in the case of degenerative sclerotic changes [34, 38, 39]. Secondly, each screw type leads to a different result so that comparability is reduced and conclusions should be drawn mainly within one study [24]. For example, Kwok et al. have measured insertion torques ranging from 0.63 ± 0.28 Nm to 1.46 ± 0.75 Nm for different screw types (in vitro, mainly osteoporotic vertebrae according DEXA, level L4 and L5) [24]. Thirdly, as also found in this study, different vertebral body levels can lead to different absolute insertion torques and screw stabilities [16, 27]. Finally, there are methodological differences (measuring instruments, pre-drilling or tapping) between the studies. However, the measured insertion torques are very plausible in comparison [16, 27]. Ozawa et al. discovered an increase in insertion torque with increasing bone density and a maximum of 1.96 Nm for normal bone density– however, their devices have been restricted to this value as a maximum, so that in comparison to the mean value of 2.3 ± 0.9 Nm from our study corresponds very well [16]. The increase in the insertion torque for caudal levels can also be confirmed [27].

One key limitation of our study is the use of frozen human cadaver specimens, which are not fully representative of the in vivo situation. However, studies have shown that such specimens have no significant detrimental influence on the results [40, 41]. Every single vertebral body included in the study exclusively possessed healthy bone density, which was difficult to obtain in large numbers. Nevertheless, this experimental study is an important step, because children’s vertebral bodies or vertebral bodies with scoliosis are not sufficiently available for testing, so that the fifty specimens included here represent a very reasonable compromise for answering the question. After the pedicle screws have been placed, the vertebral bodies have been additionally embedded on the cranial side and have undergone mechanical testing. The distraction test used does not include dynamic testing but simulates the clinical situation of the cranio-caudal loading direction of pedicle screws by a bending force that is applied to the screw head as an alternative to the conventional pullout tests [28, 29, 37, 42, 43]. This is the meanwhile preferred test method and mimics distraction procedures during scoliosis surgery as well as the force-action direction of many scoliosis implants, e.g. the ApiFix system [26, 28, 29, 37, 42–44]. So, we conclude that our test procedure reflects well the primary stability during inter-operative distractions. However, long-term effects of screw loosening due to bone remodeling (stress shielding), osteolysis as a result of wear debris, bone micro fractures because of over load, pedicle fractures, or infections could not be reproduced in vitro [4, 45]. Comparable distraction forces obtained at a 1 mm cranio-caudal displacement of a pedicle screw head are difficult to find in the literature, which emphasizes the relevance of the present study. Bianco et al. provide a rough reference value of 200 N for cadaveric lumbar vertebra with osteopenia [32] and we measured values in the range of 324.8 ± 84.4 N for healthy bone density in this study.

As a proof-of-concept example, the following formulae, (1) and (2), can be calculated from the regression data for the screws implanted here. Using the formulae, one can calculate the distraction force for a certain insertion torque and vice versa:

1. force = 165 *N* + 70 N/Nm*torque

or.

(2) torque = (force–165 N)/(70 N/Nm).

For example, assuming a child weighing 20 kg (subject to a gravitational force of 9.8 m/s²), the required minimum insertion torque for a distraction of 1 mm with forces up to 196 N is calculated to be 0.44 Nm. The statistical analysis further indicates that including screw size and vertebral level could enhance prediction accuracy.

It is important to note that this study did not methodologically assess the potential reduction in radiation exposure through the torque-based assessment of screw stability. However, this approach may present an opportunity to reduce the need for fluoroscopy during spinal surgeries. Given the high radiation exposures associated with spinal surgery [46, 47], particularly for surgeons during fluoroscopically-assisted pedicle screw insertions [48], this method could help minimize radiation risks, possibly even for pediatric patients who are more sensitive to such exposures [15, 46, 49, 50]. While further studies are required to explore the broader clinical impact and to refine this method for real-world applications, these findings offer valuable insights for improving surgical planning, implant design, and screw loosening risk prediction.

## Conclusion

This study was able to show for non-osteoporotic vertebrae that the insertion torque and distraction force necessary for a 1 mm cranio-caudal displacement of a pedicle screw head are linearly related and that the insertion torque could be used as a predictive instrument. At the same time, it was shown that vertebral level, screw geometry and rotations have an influence on the distraction force and need to be considered in future applications. In contrast, it was shown that the bone density in non-osteoporotic bone has no influence on the insertion torque and the distraction force and therefore probably plays no role on the stability of pedicle screws in healthy bone. These findings may help to further improve surgical techniques.

## Electronic supplementary material

Below is the link to the electronic supplementary material.

Supplementary Material 1: Table S1. Supplementary material. Raw data of the experiments.

## Data Availability

The data reported in this study are accessible in the supplementary material and upon reasonable request from the corresponding author, H-JW.

## Abbreviations

BMD: Bone mineral density
PMMA: Polymethylmethacrylate
qCT: Quantitative computed tomography
DEXA: Dual-energy X-ray absorptiometry
